# Combining Calcitonin and Procalcitonin and Rheumatoid Arthritis-Related Biomarkers Improve Diagnostic Outcomes in Early Rheumatoid Arthritis

**DOI:** 10.1155/2021/6331994

**Published:** 2021-05-26

**Authors:** Yingwen Liu, Jing Shi, Bo Wang, Lijing Zhou, Xiaolan Zhou, Yane Du, Dandan Li, Liang Duan, Qin Hu, Weixian Chen, Pu Li

**Affiliations:** ^1^Department of Laboratory Medicine, Peoples Hospital of Deyang City, Deyang 618000, China; ^2^Department of Laboratory Medicine, The First Hospital of Chongqing Medical University, Chongqing 400042, China; ^3^Department of Laboratory Medicine, The Second Hospital of Chongqing Medical University, Chongqing 400010, China; ^4^Department of Medical Records Section, The Second Hospital of Chongqing Medical University, Chongqing 400010, China

## Abstract

**Objective:**

To demonstrate whether procalcitonin (PCT) combined with calcitonin (CT) could provide additional diagnostic value to other clinically available rheumatoid arthritis- (RA-) related biomarkers in the early diagnosis of RA.

**Method:**

The blood samples aseptically collected by venipuncture were centrifuged within 1 hour and frozen at -80°C. PCT and CT levels were measured using electrochemiluminescence immunoassay (ECLIA) in 260 subjects (48 patients with early RA, 34 patients with established RA, 37 patients with systemic lupus erythematosus, 30 with osteoarthritis, 31 with gouty arthritis, and 80 healthy participants). Anti-cyclic citrullinated peptide (Anti-CCP) and anti-RA33 antibodies (Anti-RA33) were analyzed by ELISA. RF was detected by transmission immunoturbidimetry. Mann–Whitney *U* tests and Kruskal-Wallis tests compared differences among groups. Spearman's rank correlation analysis determined the relationship between biomarkers. Receiver-operator characteristic (ROC) curves were generated, and diagnostic performance was assessed by area under the curve (AUC), as well as specificity, sensitivity, likelihood ratios (LR).

**Results:**

Median serum PCT concentrations were significantly higher (*p* < 0.0001) in patients with early RA (0.065 ng/ml) when compared with healthy controls (0.024 ng/ml), and patients with osteoarthritis (0.025 ng/ml). When compared with gouty arthritis (GA) controls (0.072 ng/ml) and systemic lupus erythematosus (SLE) controls (0.093 ng/ml), median serum PCT concentrations were not significant in patients with early RA (0.065 ng/ml). Median serum CT concentrations were significantly lower (*p* < 0.0001) in patients with early RA (0.880 pg/ml) compared with healthy controls (3.159 pg/ml), patients with SLE (2.480 pg/ml), and patients with GA (2.550 pg/ml). When compared with osteoarthritis controls (0.586 pg/ml), median serum CT concentrations were not significant in patients with early RA (0.880 pg/ml). ROC curve analysis comparing early RA with healthy controls demonstrated that the AUC of RF, anti-CCP, and anti-RA33 were 0.66, 0.73, and 0.64, respectively; the additions of PCT and CT further improved the diagnostic ability of early RA with the AUC of 0.97, 0.98, and 0.97, respectively (*p* < 0.01). The sensitivities of RF, anti-CCP, and anti-RA33 for early RA were 33.33%, 44.74%, and 58.33%, respectively, and the additions of PCT and CT showed very high sensitivities of 83.33%, 92.11%, and 87.50%. The high-value groups of PCT moderately correlated with the anti-RA33 levels (*r* = 0.417, *p* < 0.05). CT had no significant correlation with disease duration, radiographic progression, or clinical/serological variables, such as ESR levels, CRP levels, RF, anti-CCP, and anti-RA33 levels in early RA.

**Conclusions:**

Serum PCT and CT combined with clinically available RA-related biomarkers could further improve the diagnostic efficiency of early RA.

## 1. Introduction

Rheumatoid arthritis (RA) is the most common chronic, systemic inflammatory arthritis, manifested by inflammation of synovial joints with progressive joint destruction which ultimately leads to chronic pain, bone erosions, and progressive functional disability [1]. It affects approximately 1% of the world population [2]. Once joint damage develops to extra-articular destruction (like rheumatoid nodules or vasculitis), mild symptoms may develop the severe systemic disease. In the last 5-10 years, there was growing evidence that early treatment and intervention are critical in preventing joint destruction, and as a result, it is significant to diagnose RA in the early course of the disease. Currently, the clinical diagnosis of RA mainly depends on joint involvement, acute-phase reactants, symptom duration, and serological indicators, including rheumatoid factor (RF), anti-cyclic citrullinated peptide antibody (anti-CCP), and anti-RA33 [3]. However, the 2010 criteria remain insufficient in clinical practice, especially the serological indicators. Most important of all, the sensitivity or specificity of serological indicators is limited. The meta-analysis studies report its low sensitivity (67% for anti-CCP vs. 69% for RF) and high specificity (95% for anti-CCP vs. 85% for RF). But even though its high specificity, both, RF and anti-CCP, are still detectable in patients with other rheumatic diseases, infections, as well as in apparently healthy individuals [4]. In the meta-analysis of anti-RA33 antibodies for diagnosing RA, sensitivity and specificity of anti-RA33 were 31.8% and 90.1%, respectively, indicating that anti-RA33 antibodies were highly specific but not sensitive for diagnosing RA [5]. In addition, some of the criteria, like radiographic changes, are not precise quantitative indices and mainly rely on the subjective judgment of rheumatologists. Therefore, it is crucial to find significant diagnostic tools like additional serum biomarkers that improve sensitivity for the diagnosis of RA while maintaining high specificity.

Calcitonin (CT), a 32-amino-acid monomeric peptide physiologically produced mainly from the thyroid C-cells, results from cleavage and posttranslational processing of procalcitonin (PCT) [6]. CT is widely used in the clinical treatment of osteoporosis. In recent years, more and more studies have indicated that CT can prevent bone resorption, promote the synthesis of chondrocytes and extracellular matrix, and inhibit cartilage degradation [7]. CT inhibits the degeneration of articular cartilage by inhibiting the activation of toll-like receptors (TLR) and the production of endogenous ligands.

PCT, a 116-amino-acid precursor protein, is usually generated and cleaved to CT in the CT cells of the thyroid gland [8, 9]. RA is characterized by the presence of varying degrees of systemic inflammation occurring besides local joint inflammation. Soluble inflammatory mediators of systemic inflammation such as C-reactive protein (CRP), interleukin-6 (IL-6), tumor necrosis factor (TNF-*α*), and anti-inflammatory marker IL-10 are highly expressed in synovium fluid and serum of arthritic patients, which play a critical role in the pathophysiology of RA [10, 11]. Some reports have mentioned that in systemic inflammation, PCT is released from various forms of proinflammatory cytokines, such as IL-6, TNF-*α*, and interleukin-1 (IL-1) [12].

The activation of TLR is a double-edged sword, which is the cause of cancer, autoimmune diseases, chronic inflammation, and neurodegenerative diseases [13]. Degradation products after tissue injury, such as heat shock proteins and extracellular matrix decomposition products (such as hyaluronic acid fragments, heparan sulfate, fibrinogen, fibronectin extra domain, and high-mobility group protein 1) can be used as endogenous ligands to activate TLR [14]. As mentioned above, in the process of osteoarthritis, TLRs binding with endogenous ligands can release a large number of cytokines and inflammatory mediators, including TNF, IL-1, IL-6, IL-8, and MMPs, which further aggravate synovitis and cartilage damage [15]. Therefore, we speculated that in the early stage of RA, on the one hand, the activation of TLR releases a large number of inflammatory mediators that lead to the increase of PCT levels. On the other hand, the generation of endogenous ligands and the activation of TLR, in turn, inhibit the production of CT and lead to a decrease of CT levels. However, the role of PCT and CT in the early diagnosis of RA is unclear. We aim to demonstrate whether the measurement of PCT and CT has diagnostic value in early RA and whether PCT combined with CT could provide additional diagnostic value to other clinically available RA-related biomarkers in the early diagnosis of RA.

## 2. Materials and Methods

### 2.1. Patients and Controls

Cases and healthy controls were retrospectively collected from October 2018 to November 2019 by reviewing the electronic medical records. According to the study of the clinical charts, included patients were classified into 3 groups. On a total of 180 patients, 82 patients were diagnosed with RA, according to the 2010 RA Classification Criteria by the American College of Rheumatology (ACR), and 98 patients were classified as non-RA. The RA group was subdivided according to disease duration in the early RA group with disease duration ≤1 year and established RA group with disease duration >1 year. The non-RA group included 37 patients diagnosed with SLE, 30 with OA, and 31 with GA. The last group included 80 healthy individuals. All patients' medical records were reviewed, and the relevant clinical and serological data were collected. The study was endorsed by the local ethics committee, and each patient provided informed consent before entering the study.

### 2.2. Laboratory and Clinical Assessments

Serum samples were collected from the patients on medical visits to our hospital and taken aseptically by venipuncture and centrifuged within 1 h. The samples were stored frozen at -80°C until further analysis. CT and PCT were measured on a Roche Cobas e601 system (BRAHMS, Berlin, Germany) by ECLIA. The assay has a functional sensitivity of 0.06 ng/ml. RF was detected by Roche Cobas c311 system (BRAHMS, Berlin, Germany) with a range of 10-130 IU/ml, and cutoff set at 14 IU/ml. Anti-CCP and was analyzed by a commercial ELISA kit (YHLO, Shenzhen, China) and considered positive at a cutoff value of 30 AU/ml. Anti-RA33 was monitored using a commercial ELISA kit (YHLO, Shenzhen, China), and normal ranges were 0 to 25 AU/ml for anti-RA33.

### 2.3. Statistical Analysis

We performed a statistical analysis that compared the general information of each group. Qualitative items were presented as numbers and percentages for the description of the basic characteristics, and quantitative items were identified as means ± standard deviation or medians (upper and lower quartile). The Shapiro-Wilk method was conducted to test whether the data were normally distributed, and the Levene method was used to test the homogeneity of variance. Because our data did not meet the criteria for normal distribution, the non-parametric Mann–Whitney *U* and Kruskal-Wallis tests were conducted for comparisons of continuous variables across different groups. Unconditional logistic regression analysis was used to examine whether each variable was an independent factor in RA. Spearman's rank correlation analysis was carried out to determine the relationship between biomarkers. A *p* < 0.05 denoted statistical significance, and the results were described as odds ratio with 95% confidence intervals (CIs).

In order to determine whether there is an advantage in diagnosing RA when using both tests compared to using a single RA biomarker, we undertook a ROC (receiver operating characteristic) analysis and calculated the areas under the curve (AUC). The areas under ROC curves were estimated by the nonparametric method of Mann–Whitney statistics. We evaluated the detailed diagnostic performance of PCT and CT combined with the present biomarkers of RA according to the AUC, sensitivity, specificity, positive likelihood ratios (LR), and negative likelihood ratios (LR). Confidence intervals (95%) for the AUC were performed in Medcalc statistical software version 15.2.2 (MedCalc Software bvba, Ostend, Belgium). In all analyses, a *p* < 0.05 denoted statistical significance, and the results were described as the odds ratio with 95% confidence intervals (CIs). The statistical analyses for quantitative variables were performed using SPSS version 22.0 (SPSS, Chicago, IL).

## 3. Results

### 3.1. Study Population Demographics

A total of 260 patients were involved. In RA, 48 cases had early RA, 34 of them (70.8%) were females, and 34 cases with established RA aged between 44 and 84 years, 32 of them (94.1%) were females. In non-RA, 37, 30, and 31 patients had SLE, OA, and GA, respectively. The corresponding male/female ratios were 3/34, 4/26, and 30/0, respectively, and the corresponding average ages were 43 ± 11, 52 ± 14, and 53 ± 16, respectively. The 80 healthy controls included 42 females (52.5%), and the corresponding average ages were 50 ± 13. Demographic data, some clinical manifestations, joint-space narrowing score, erosion score, and laboratory data were summarized in Table 1.

For normally distributed data, values were expressed as mean ± SD; data not distributed normally expressed as median (range). RA: rheumatoid arthritis; SLE: systemic lupus erythematosus; OA: osteoarthritis; GA: gouty arthritis; CRP: C-reactive protein; ESR: erythrocyte sedimentation rate.

### 3.2. The Levels of PCT and CT Were Significantly Different in Patients with Early Rheumatoid Arthritis than in those Control Groups

**Figure 1:**
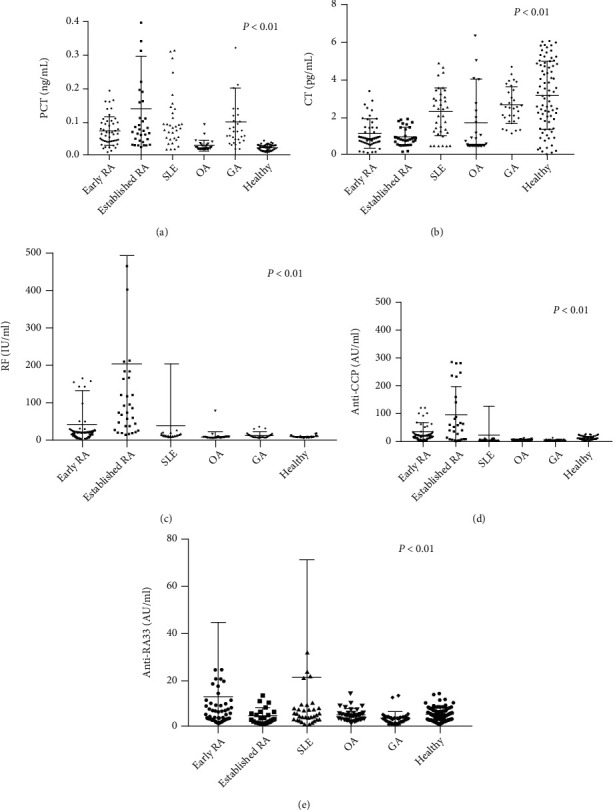
(a) Distribution of the serum PCT level in early RA, established RA, SLE, OA, GA, and healthy control groups. (b) Distribution of the level of serum PCT in early RA, established RA, SLE, OA, GA, and healthy control groups. (c) Distribution of the serum RF level in early RA, established RA, SLE, OA, GA, and healthy control groups. (d) Distribution of the serum anti-CCP level in early RA, established RA, SLE, OA, GA, and healthy control groups. (e) Distribution of the serum anti-RA33 level in early RA, established RA, SLE, OA, GA, and healthy control groups. RA: rheumatoid arthritis; SLE: systemic lupus erythematosus; OA: osteoarthritis; GA: gouty arthritis; CRP: C-reactive protein; ESR: erythrocyte sedimentation rate; CT: calcitonin; PCT: procalcitonin; anti-CCP: anti-cyclic citrullinated peptide; anti-RA33: anti-RA33 antibodies.

A Mann–Whitney *U* test was run to determine if there were differences in those biomarkers between early RA patients, established RA patients, non-RA patients, and healthy individuals (Figure 1). As shown in Figure 1(a), among the biomarkers, differences in the median serum level of PCT were statistically significant between the early RA (0.065 ng/ml) and OA groups (0.025 ng/ml) (*p* < 0.0001) or between the RA (0.065 ng/ml) and healthy groups (0.024 ng/ml) (*p* < 0.0001). When compared with GA controls (0.072 ng/ml) and SLE controls (0.093 ng/ml), median serum PCT concentrations were not significant in patients with early RA (0.065 ng/ml). The concentrations of CT were found to be significantly different in early RA patients as compared with the SLE, GA, and healthy groups (*p* < 0.0001), and upon further investigation, it was determined that this difference in expression resided mostly between healthy individuals and patients with early RA: median serum CT concentrations were significantly lower (*p* < 0.0001) in patients with early RA (0.880 pg/ml) compared with healthy controls (3.159 pg/ml) as presented in Figure 1(b). Other laboratory measurements were also investigated, such as RF, anti-CCP, and anti-RA33. The levels of RF and anti-CCP were not significant in the early RA, while the established RA patients, as a group, had significantly increased levels of RF and anti-CCP compared with non-RA groups and healthy controls (*p* < 0.0001). There were no significant differences in the concentration of anti-RA33 between the early RA, established RA, non-RA, and healthy control groups (Figure 1). As shown in Table 2, median serum PCT concentrations had an increasing trend from early RA to established RA. On the contrary, median serum CT concentrations decreased in early RA and had a decreasing trend from early RA to established RA.

The data was divided into 5 groups. Data are expressed as medians. RA: rheumatoid arthritis; SLE: systemic lupus erythematosus; OA: osteoarthritis; GA: gouty arthritis; PCT: procalcitonin; CT: calcitonin; RF: rheumatoid factor; anti-CCP: anti-cyclic citrullinated peptide; anti-RA33: anti-RA33 antibodies.

### 3.3. Associations between PCT, CT, and Clinical Features in Early RA

The correlation matrix provided in Tables 3 and 4 illustrated the relationship between the PCT levels, CT levels, radiographic progression, and clinical/serological variables in the early RA cohort and the entire course of the RA cohort. Spearman's rank correlation analysis found that no significant correlation was observed between serum levels of PCT and ESR levels, CRP levels, RF, anti-CCP, and anti-RA33 levels in early RA. Meanwhile, serum CT levels did not correlate with ESR levels, CRP levels, RF, anti-CCP, or anti-RA33 levels in early RA. In the entire course of RA, spearman's rank correlation analysis found that serum levels of PCT moderately correlated with the ESR (*r* = 0.360, *p* < 0.05), CRP (*r* = 0.371, *p* < 0.05). Moreover, no significant correlation was observed with RF, or between levels of anti-CCP, and anti-RA33. Besides, serum CT levels did not correlate with ESR levels, CRP levels, RF, anti-CCP, or anti-RA33 levels. In the cases of RA, we calculated the correlation of serum PCT and CT with disease duration. No significance was observed whether in the early course of the disease or in the whole course of the disease. Regarding radiographic progression, the Sharp scores of RA patients were calculated, and we assessed the correlation of serum PCT and CT with the Sharp scores. However, it showed no significant statistical correlation of serum PCT and CT with radiological assessment results whether in the early course of the disease or in the whole course of the disease.

All correlations were established with a Spearman rank correlation as variables were nonnormally distributed according to Shapiro-Wilk normality testing. PCT: procalcitonin; CT: calcitonin; ESR: erythrocyte sedimentation rate; CRP: C-reactive protein; RF: rheumatoid factor; anti-CCP: anti-cyclic citrullinated peptide; anti-RA33: anti-RA33 antibodies; ^∗^*p* < 0.05.

All correlations were established with a Spearman rank correlation as variables were nonnormally distributed according to Shapiro-Wilk normality testing. PCT: procalcitonin; CT: calcitonin; ESR: erythrocyte sedimentation rate; CRP: C-reactive protein; RF: rheumatoid factor; anti-CCP: anti-cyclic citrullinated peptide; anti-RA33: anti-RA33 antibodies; ^∗^*p* < 0.05.

**Figure 2:**
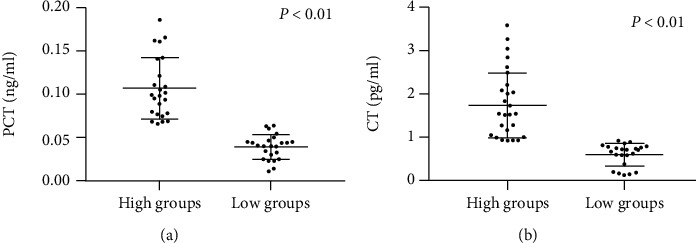
(a) Distribution of the serum PCT level in high-value groups and low-value groups. (b) Distribution of the serum CT level in high-value groups and low-value groups. PCT: procalcitonin; CT: calcitonin.

Furthermore, as shown in Figure 2, we divided the early RA patients into the high-value groups and the low-value groups of PCT and CT using the best cut-off value of each variable. Spearman's rank correlation analysis provided the relationship between the high-value groups and low-value groups of PCT and CT, disease duration, and clinical/serological variables in the early RA cohort. As shown in Table 5, Spearman's rank correlation analysis found that the high-value groups of PCT moderately correlated with the anti-RA33 levels (*r* = 0.417, *p* < 0.05). Moreover, no significant correlation was observed with disease duration, or between levels of RF and anti-CCP. Besides, as shown in Table 6, both the high-value groups of CT and the low-value groups of CT did not correlate with disease duration, RF, anti-CCP, or anti-RA33 levels.

All correlations were established with a Spearman rank correlation as variables were nonnormally distributed according to Shapiro-Wilk normality testing. PCT: procalcitonin; RF: rheumatoid factor; anti-CCP: anti-cyclic citrullinated peptide; anti-RA33: anti-RA33 antibodies; ^∗^*p* < 0.05.

All correlations were established with a Spearman rank correlation as variables were nonnormally distributed according to Shapiro-Wilk normality testing. PCT: procalcitonin; RF: rheumatoid factor; anti-CCP: anti-cyclic citrullinated peptide; anti-RA33: anti-RA33 antibodies; ^∗^*p* < 0.05.

### 3.4. Additions of Serum PCT and CT Assay Improve the Diagnostic Performance of RF, anti-CCP, and anti-RA33 in early RA

ROC analysis was conducted on a single serum biomarker and combinations of serum biomarkers, and we found an impressive additional diagnostic value of PCT and CT compared with the single use of the present biomarkers alone. We compared early RA patients with controls including all controls, disease controls (SLE groups, OA groups, and GA groups), and healthy individuals.

**Figure 3:**
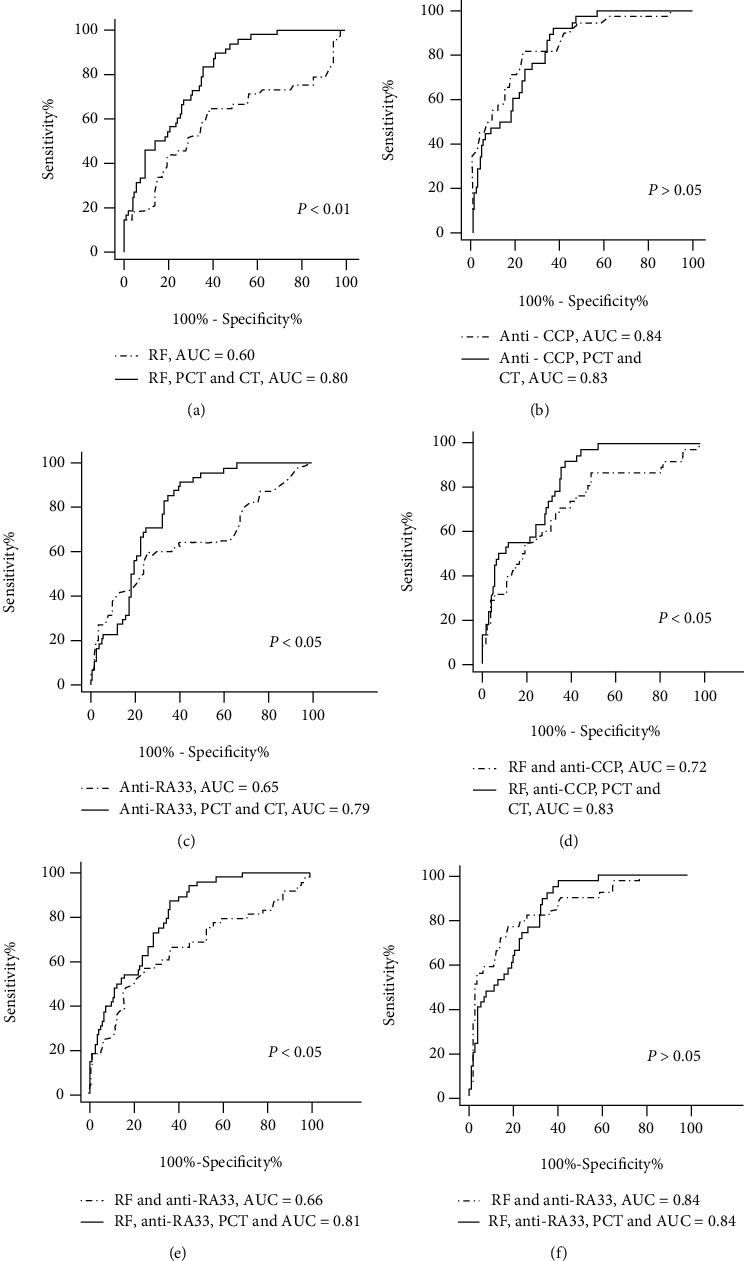
Corresponding ROC curve for a single serum biomarker and combinations of serum biomarkers in early rheumatoid arthritis along with their respective area under the curve (AUC), comparing with all controls. (a) ROC analysis of RF, RF combined with PCT and CT. (b) ROC analysis of anti-CCP, anti-CCP combined with PCT and CT. (c) ROC analysis of anti-RA33, anti-RA33 combined with PCT and CT. (d) ROC analysis of RF and anti-CCP, RF and anti-CCP combined with PCT and CT. (e) ROC analysis of RF and anti-RA33, RF and anti-RA33 combined with PCT and CT. (f) ROC analysis of anti-CCP and anti-RA33, anti-CCP and anti-RA33 combined with PCT and CT.

As shown in Figure 3, when early RA patients compared with all controls, the AUC of single indicators of RF and anti-RA33 was 0.60 and 0.65, respectively, while with the additions of PCT and CT, the diagnostic ability for early RA further improved with the AUC of 0.80 and 0.79, respectively (*p* < 0.05). The AUC of single indicators of anti-CCP decreased with the additions of PCT and CT, which was not statistically significant. Furthermore, we evaluated the ROC curve using a combination of serum biomarkers for diagnosis. In our study, the AUC of the combination of RF and anti-CCP raised from 0.72 to 0.83 with the additions of PCT and CT (*p* < 0.05). And the AUC of the combination of RF and anti-RA33 significantly increased from 0.66 to 0.81 with the additions of PCT and CT (*p* < 0.05). However, the AUC of the combination of anti-CCP and anti-RA33 with the additions of PCT and CT had no significant change, which was not statistically significant.

**Figure 4:**
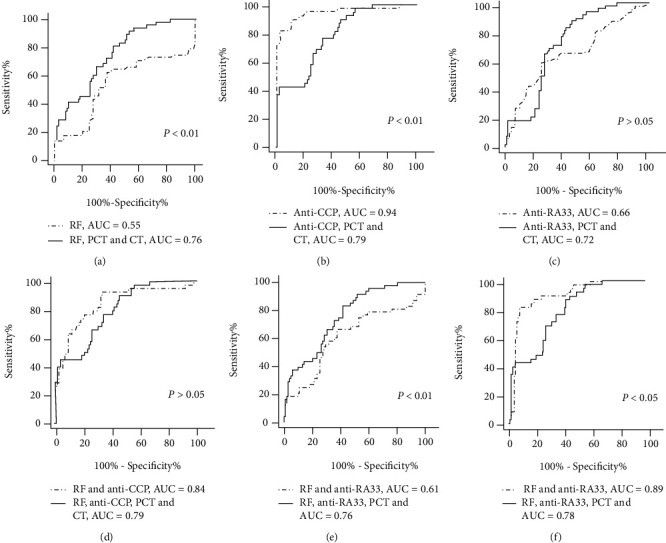
Corresponding ROC curve for a single serum biomarker and combinations of serum biomarkers in early RA along with their respective area under the curve (AUC), compared with disease controls (SLE groups, OA groups, and GA groups). (a) ROC analysis of RF, RF combined with PCT and CT. (b) ROC analysis of anti-CCP, anti-CCP combined with PCT and CT. (c) ROC analysis of anti-RA33, anti-RA33 combined with PCT and CT. (d) ROC analysis of RF and anti-CCP, RF and anti-CCP combined with PCT and CT. (e) ROC analysis of RF and anti-RA33, RF and anti-RA33 combined with PCT and CT. (f) ROC analysis of anti-CCP and anti-RA33, anti-CCP and anti-RA33 combined with PCT and CT.

As shown in Figure 4, when early RA patients compared with disease controls, we found that only the AUC of single indicators of RF increased with the additions of PCT and CT, which from 0.55 to 0.76 (*p* < 0.01). While the AUC of single indicators of anti-CCP decreased with the additions of PCT and CT, which from 0.94 to 0.79 (*p* < 0.01). The AUC of the anti-RA33 single index was raised with the additions of PCT and CT, but the difference was not statistically significant. Similarly, we assessed the ROC curve for diagnosis with a combination of serum biomarkers. We found that only the AUC of the combination of RF and anti-RA33 increased from 0.61 to 0.76 with the additions of PCT and CT (*p* < 0.01). While with the addition of PCT and CT, the AUC of anti-CCP combined with anti-RA33 decreased from 0.89 to 0.78 (*p* < 0.05). At last, the AUC of RF and anti-CCP combined with PCT and CT decreased from 0.84 to 0.79, but the difference was not statistically significant.

**Figure 5:**
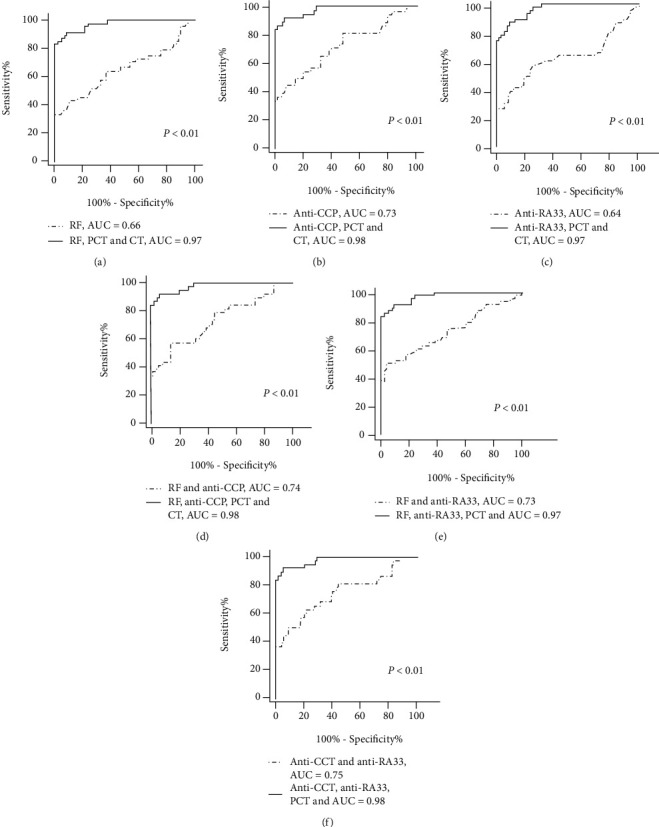
Corresponding ROC curve for a single serum biomarker and combinations of serum biomarkers in early rheumatoid arthritis along with their respective area under the curve (AUC), compared with healthy individuals. (a) ROC analysis of RF, RF combined with PCT and CT. (b) ROC analysis of anti-CCP, anti-CCP combined with PCT and CT. (c) ROC analysis of anti-RA33, anti-RA33 combined with PCT and CT. (d) ROC analysis of RF and anti-CCP, RF and anti-CCP combined with PCT and CT. (e) ROC analysis of RF and anti-RA33, RF and anti-RA33 combined with PCT and CT. (f) ROC analysis of anti-CCP and anti-RA33, anti-CCP and anti-RA33 combined with PCT and CT.

As shown in Figure 5, when early RA patients compared with healthy individuals, we found impressive differences with the additions of PCT and CT compared with the single indicators. In our study, the AUC of RF, anti-CCP, and anti-RA33 was 0.66, 0.73, and 0.64, respectively, while with the additions of PCT and CT, the diagnostic ability of early RA was further improved, and the AUC was 0.97, 0.98, and 0.97, respectively (*p* < 0.01). At last, we investigated the roc curve for diagnosis in combination with serum biomarkers. We found that with the additions of PCT and CT, the combination of RF and anti-CCP, RF and anti-RA33, anti-CCP and anti-RA33 significantly increased from 0.74, 0.73, and 0.75 to 0.98, 0.97, and 0.98, respectively (*p* < 0.05).

The sensitivity, specificity, positive likelihood ratios (LR), and negative likelihood ratios (LR) of RF, anti-CCP, and anti-RA33 with and without PCT and CT in early RA patients were summarized in Table 7. ROC curves were analyzed to define a cut-off value for the highest sensitivity and specificity of predicted probabilities of RF, anti-CCP, and anti-RA33 with and without PCT and CT in early RA patients, which achieved *p* ≤ 0.05 using a Mann–Whitney *U* test. In the early RA group compared with all controls, the additions of PCT and CT led to notable increases in sensitivity without substantial loss of specificity. In the RF comparison group, there were substantial increases in sensitivity with the additions of PCT and CT, with only a modest decrease in specificity. The ROC curve of RF yielded a sensitivity of 64.58% and a specificity of 61.24%, and the additions of PCT and CT increased sensitivity to 89.58% and specificity to 58.99% (*p* < 0.01). In the anti-RA33 comparison group, the ROC curve demonstrated a sensitivity of 58.33% and a specificity of 74.72%, and the additions of PCT and CT were reported to have a sensitivity of 91.67% and a specificity of 59.55% (*p* < 0.01), which was significantly different from the single anti-RA33 group. In the RF combined with the anti-CCP group, the additions of PCT and CT led to significant increases in sensitivity with modest increases of specificity. The ROC curve of RF combined with anti-CCP indicated a sensitivity of 86.84% and a specificity of 50.56%, and the additions of PCT and CT increased sensitivity to 92.11% and specificity to 62.36% (*p* < 0.01). In our study, the combination of RF and anti-CCP with the additions of PCT and CT is the most effective method in the diagnosis of early RA. According to the Youden Index, defined as sensitivity + specificity -1, the best cutoff value of prediction probability of the proposed model was 0.439, with a sensitivity and specificity of 92.11% and 62.36%, respectively (AUC = 0.83, 95% CI: 0.768 to 0.874, *p* < 0.01).

In the early RA group compared with disease controls, only the RF comparison group and the RF combined with the anti-RA33 group demonstrated to have substantial increases in sensitivity and only a modest decrease in specificity with the additions of PCT and CT. The sensitivities of the RF group and the RF combined with the anti-RA33 group were 64.58% and 66.67%, respectively, and the specificities were 60.20% and 62.24%, respectively. The additions of PCT and CT yielded high sensitivities of 81.25% and 83.33% and specificities of 58.16% and 58.16% (*p* < 0.01). When the predicted probability of the combination of RF and anti-RA33 with the additions of PCT and CT was plotted in a ROC curve (AUC = 0.76, 95% CI: 0.681 to 0.826, *p* < 0.01), the best cutoff obtained was 0.438, with a sensitivity = 83.33% and specificity = 58.16%.

In the early RA group compared with healthy controls, the additions of PCT and CT led to significant increases in sensitivity and specificity in all comparison groups. The sensitivities of RF, anti-CCP, and anti-RA33 for early RA were 33.33%, 44.74%, and 58.33%, respectively, and the specificities were 100.00%, 92.50%, and 75.00%. The additions of PCT and CT showed very high sensitivities of 83.33%, 92.11%, and 87.50% and modest specificities of 100.00%, 93.75%, and 91.25%. In addition, the combination of RF and anti-CCP, RF and anti-RA33, anti-CCP, and anti-RA33 showed a sensitivity of 57.89%, 50.00%, and 63.16% and a specificity of 86.25%, 96.25%, and 78.75%. The additions of PCT and CT showed very high sensitivities of 92.11%, 83.33%, and 92.11% and specificities of 93.75%, 100.00%, and 95.00%. Taking all factors together suggests that the combination of anti-CCP and anti-RA33 with the additions of PCT and CT is the most effective method in the diagnosis of early RA, which showed a sensitivity of 92.11% and a specificity of 95.00% (*p* < 0.01). The predicted probability of the combination of anti-CCP and anti-RA33 with the additions of PCT and CT achieved the highest performance and a cut-off value equal to 0.296 yielded to the best diagnostic results (AUC = 0.98, 95% CI: 0.931 to 0.996, *p* < 0.01).

## 4. Discussion

RA is a chronic systemic inflammatory disease of unclear etiology that is characterized by a progressive and destructive polyarthritis in company with serological evidence of autoreactivity. It is manifested by chronic pain and joint destruction, usually progressing from the distal end to the proximal joints [16]. It is widely acknowledged that the critical factors in the prevention of joint damage are early detection of RA and therapeutic intervention [17]. In recent years, it becomes more and more evident that early treatment and intervention at a very early stage of the disease bring more effective disease control, less joint destruction, and better prognosis of the disease [18–20]. Therefore, the early and accurate diagnosis has become more and more valuable, and biological markers which support an early diagnosis are of great significance to improve disease outcome. But the diagnosis of RA could be challenging, particularly in early disease and in patients with atypical performance [21]. The clinical manifestations of RA and other arthritis in the early stages of the disease are not always characteristic; moreover, classification criteria for the established RA are usually not met at an early stage [22, 23]. About one-third of the patients with persistent arthritis do not meet the classification criteria, so it is frequently hard to diagnose RA at a very initial stage of the disease [24, 25]. Due to the relatively poor sensitivity of conventional biomarkers, there is a need for additional serum biomarkers which could effectively improve sensitivity for the diagnosis of RA while maintaining high specificity. In the recent study, we demonstrated that PCT and CT in combination with other clinically available RA-related biomarkers could improve the diagnostic performance of early RA.

One of the most crucial problems in RA diagnosis is the absence of sensitivity or specificity of the current indicators, anti-citrullinated protein antibodies (ACPA), and RF. Moreover, ACPA is an overlapping group of antibodies depending on the citrullination of an arginine residue, which includes antiperinuclear factor (APF) [26], antikeratin antibody (AKA) [27], anti-filaggrin antibodies (AFA) [28], anti-Sa [29], and anti-CCP antibodies [30, 31], and only anti-CCP antibody is used in clinical practice. By using a single CCP as the antigen in an ELISA test, the anti-CCP antibody is as sensitive as RF in RA and more specific than RF. However, the sensitivity and specificity reported in the different studies rely on the cutoff titer selected for the positive test, the characteristics of the arthritis population using the test, and the gold criteria selected for RA; they range from 30% to 70% and 91% to 99%, respectively [32, 33]. RF fares even worse, with specificity ranging from 38% to 85%, suggesting that positive IgM-RF has a moderate diagnostic value [4, 34]. However, in our study, the sensitivities of RF and anti-CCP for early RA were 33.33% and 44.74%, respectively, and the specificities were 100.00% and 92.50%. The additions of PCT and CT showed very high sensitivities of 83.33% and 92.11% and modest specificities of 100.00% and 93.75%. Because of the high specificity of ACPA and the relatively high sensitivity of RF, it is advisable to combine RF with ACPA to diagnose RA. This combination does improve the diagnostic value of these tests. However, there are studies indicating that even the combination of these two indicators is not very excellent. The sensitivity and specificity of RF- or ACPA-positive patients are 78% and 82%, respectively [35]. In our study, the combination of RF and anti-CCP showed a sensitivity of 57.89% and a specificity of 86.25%. However, the additions of PCT and CT showed a very high sensitivity of 92.11% and a specificity of 93.75%. On the other hand, varieties of circulating non-RF antibodies have been found and reported having potential diagnostic values during the last years. However, most of these autoantibodies could not prove to be sensitive and specific enough to form a basis for clinical and therapeutic decisions, including ANA, antiperinuclear antibodies, antikeratin antibodies, and anti-Sa antibodies [36–38].

The first important finding from our data was that the serum PCT significantly increased (*p* < 0.0001) in early RA patients (0.065 ng/ml) compared with healthy controls (0.024 ng/ml) and the serum CT concentrations were significantly lower (*p* < 0.0001) in patients with early RA (0.880 pg/ml) compared with healthy controls (3.159 pg/ml). This difference might be explained by different levels of inflammation, different treatments, and interventions in RA patients among different researches, which needed further study to be investigated.

Our study was designed to evaluate not only the clinical utility of single indicators such as RF, anti-CCP, and anti-RA33 in RA but also the physical effect of every possible combination with the additions of PCT and CT, among RF, anti-CCP, and anti-RA33. The result of the ROC curve indicated the combinations of PCT, CT and RF, PCT, CT and anti-CCP, PCT, CT, and RA33 further improved the diagnostic ability for RA with the AUC of 0.97, 0.98, and 0.97, while the AUC of single indicators of RF, anti-CCP, and anti-RA33 was 0.66, 0.73, and 0.64, respectively. With the combination of PCT and CT, the sensitivity has risen, at a moderate cost of a decline in specificity, compared to a single biomarker. In our study, out of all evaluated combinations, it showed that the most clinically useful test was the union of anti-CCP and anti-RA33 with the additions of PCT and CT that can produce not only the highest sensitivity (92.11%) but also higher specificity (95.00%), with an AUC of 0.98. So we can draw a conclusion that the combined use of PCT and CT is a powerful diagnostic tool and shows a higher value for clinical use than the single detection of conventional indicators only.

Most of the studies have stated that the levels of serum PCT [39–41], serum CRP, and ESR [42] considerably improve the diagnostic accuracy of serum inflammatory markers in infectious arthritis. However, the role of PCT in noninfectious arthritis remains to be at the forefront. Studies have suggested that PCT can be applied in the differential diagnosis of noninfectious arthritis, such as RA, other noninfectious arthritis, and bacterial infections [43–45]. Although high levels of PCT usually occur at the time of infection, PCT levels may also increase under noninfectious conditions, including the stress associated with surgery or trauma [46] and high inflammatory states associated with certain autoimmune diseases [47–51]. RA is an inflammatory disease, and the inflammatory markers are highly expressed in synovial fluid and serum of patients with arthritis, such as C-reactive protein (CRP: an acute-phase protein), interleukin-6 (IL-6), and tumor necrosis factor (TNF-*α*). IL-6 is the most abundant cytokine expressed in patients with RA, which has the biological activities of regulating the immune response, inflammation, and hematopoiesis. TNF-*α* is one of the key proinflammatory cytokines that lead to inflammation and joint destruction in RA. Some studies have reported that PCT is released by various forms of proinflammatory cytokines in systemic inflammation, such as IL-6, TNF-*α*, and IL-1 [52]. In our study, the median serum level of PCT was statistically significant between the early RA (0.065 ng/ml) and healthy groups (0.024 ng/ml) (*p* < 0.0001). In addition, the median serum PCT concentrations had an increasing trend from early RA to established RA. Therefore, we speculated that the change of serum PCT might be related to the release of proinflammatory cytokines in early RA. Interestingly, there is no noticeable increase in CT levels in severe systemic inflammation, which may be related to the significant resistance to enzymatic degradation of CT precursors [53]. And in our study, the median serum CT concentrations were significantly lower in patients with early RA (0.880 pg/ml) compared with healthy controls (3.159 pg/ml) (*p* < 0.0001). In addition, the level of median serum CT decreased in early RA and had a decreasing trend from early RA to established RA, which was consistent with previous reports. So, we speculated on this theory that PCT and CT might participate in the mechanism of RA. However, data concerning serum PCT and CT levels on patients with active underlying systemic autoimmune diseases, inflammatory arthritis is limited. There are limited published studies regarding PCT and CT levels in patients with RA until now.

There were some negative results in our study that might contribute to the application of PCT and CT in diagnosing RA. No correlation was observed between serum concentrations of PCT, CT, and the disease duration of early RA. This indicated that the diagnostic efficiency of PCT and CT may not decrease in early RA, whereas there could be a significant decline in the sensitivity of anti-CCP when diagnosing patients with early RA rather than patients with established RA [54]. Interestingly, almost no association of PCT or CT with Sharp scores was found in our study, indicating that PCT and CT might be used without too much consideration of the extent of joint damage. Moreover, these criteria are not very effective because they can only help diagnose patients who already have severe structural destruction. In addition, some of the criteria, such as radiographic changes, are not accurate quantitative index and generally rely on the subjective judgment of rheumatologists [55]. In addition, after we divided the early RA patients into the high-value groups and the low-value groups of PCT and CT, we found that the high-value groups of PCT moderately correlated with the anti-RA33 levels (*r* = 0.417, *p* < 0.05) using Spearman's rank correlation analysis. This may indicate that the higher the PCT level, the more useful it may be to improve the efficiency of combined diagnosis in early RA.

Our study also has a few limitations. First, many studies did not report major methodological features, like the study setting, the inclusion and exclusion criteria, or disease severity. Lacking information reduced transparency of the methods and results and made it hard to exclude bias. Second, we collected the data from a single-center, so the number of RA patients was small. Consequently, there is a possibility that the results of our study could be different from those of other centers, and the predictive probability could be overestimated compared with a prospective study. In heterogeneous diseases such as RA, small sample sizes not only limit generalizability but also ignore important associations and may restrict the number of variables that could be contained in a multivariate analysis. As a result, additional prospective studies with larger populations including multiple centers are necessary in order to confirm our conclusion. Finally, we lack the follow-up data of the patients with RA. Long-term follow-up of RA patients with abnormal PCT or CT is necessary to assess the diagnostic value of this test in patients with RA.

## 5. Conclusion

In conclusion, our studies identified that the combination of PCT, CT, and clinically available RA-related biomarkers could further improve the diagnostic efficiency of RA, compared with the single use of the present biomarkers alone. In addition, we observed that the sensitivities of RF, anti-CCP, and anti-RA33 for RA significantly improved with the combination of PCT and CT. At last, we recommend that the combination of RF, anti-CCP, PCT, and CT is the most effective method in the diagnosis of early RA.

## Figures and Tables

**Table 1:** Demographic and clinical characteristics of the participants.

Characteristic	Early RA	Established RA	Non-RA			Healthy controls
			SLE	OA	GA	
Total	48	34	37	30	31	80
Age (years)	50 ± 11	63 ± 10	43 ± 11	52 ± 14	53 ± 16	50 ± 13
Females (%)	34 (70.8%)	32 (94.1%)	34 (91.9%)	26 (86.7%)	0 (0%)	42 (52.5%)
Disease duration (years)	0.9 (0.6–1.0)	6.0 (3.8–11.0)	—	—	—	—
Tender joint count	12 (4–17)	10 (5–20)	—	—	—	—
Swelling joint count	3 (0–13)	3 (1–10)	—	—	—	—
CRP (mg/L)	17.91 (5.81–45.51)	23.40 (11.11–66.43)	—	—	—	—
ESR (mm/h)	33.82 ± 28.07	49.07 ± 32.57	—	—	—	—
Radiographic progression, median						
Joint-space narrowing score	1 (1–1)	1 (1–2)	—	—	—	—
Erosion score	1 (1–2)	3 (2–4)	—	—	—	—

**Table 2:** Differences between the serum biomarkers level in the study groups.

Group	PCT, ng/ml	CT, pg/ml	RF, IU/ml	Anti-CCP, AU/ml	Anti-RA33, AU/ml
	Median (IQR)	Median (IQR)	Median (IQR)	Median (IQR)	Median (IQR)
Early RA, *n* = 48	0.065 (0.040–0.099)	0.880 (0.675–1.470)	18.8 (7.2–29.5)	20.0 (11.8–50.6)	6.6 (3.3–16.3)
Established RA, *n* = 34	0.076 (0.044–0.171)	0.845 (0.659–1.515)	90.2 (38.7–192.4)	57.8 (10.4–177.1)	3.3 (1.6–6.1)
All controls, *n* = 178	0.029 (0.021–0.060)	2.480 (1.205–3.910)	11.9 (8.8–19.7)	4.5 (3.3–10.3)	4.3 (2.9–6.2)
All disease controls, *n* = 98	0.056 (0.027–0.101)	2.110 (0.873–3.070)	12.3 (9.5–30.3)	3.6 (2.8–4.6)	4.1 (2.7–6.6)
SLE, *n* = 37	0.093 (0.054–0.169)	2.480 (1.235–3.210)	31.3 (13.2–41.3)	3.1 (2.1–5.0)	5.4 (2.9–9.4)
OA, *n* = 30	0.025 (0.020–0.031)	0.586 (0.500–2.045)	9.4 (7.5–11.1)	4.2 (3.6–4.9)	4.6 (3.1–5.9)
GA, *n* = 31	0.072 (0.045–0.120)	2.550 (1.970–3.490)	10.8 (9.2–15.8)	3.5 (2.2–4.1)	2.9 (1.9–4.1)
Healthy, *n* = 80	0.024 (0.017–0.028)	3.159 (1.757–4.827)	11.7 (6.6–18.8)	10.4 (5.8–18.5)	4.6 (3.4–6.1)

**Table 3:** Correlation coefficients of PCT and CT with clinical and serological measures in early RA.

Variable	Age	Disease duration	Sharp scores	ESR	CRP	RF	Anti-CCP	Anti-RA33	PCT
*n*	48	48	48	48	48	48	38	48	48
PCT	0.050	0.024	-0.188	0.442	0.414	-0.245	0.105	0.263	—
CT	0.033	-0.034	0.679	-0.073	0.067	-0.073	0.047	0.070	0.092

**Table 4:** Correlation coefficients of PCT and CT with clinical and serological measures in the whole course of RA.

Variable	Age	Disease duration	Sharp scores	ESR	CRP	RF	Anti-CCP	Anti-RA33	PCT
*n*	82	82	82	82	82	82	64	71	82
PCT	0.064	0.183	0.025	0.360∗	0.371∗	0.063	0.223	0.090	—
CT	-0.132	0.009	0.039	-0.097	0.232	-0.015	0.014	0.194	0.205

**Table 5:** Correlation coefficients of the high-value groups of PCT with clinical and serological measures in early RA.

Variable	Disease duration	RF	Anti-CCP	Anti-RA33
Low groups of PCT	0.292	-0.215	0.006	0.085
High groups of PCT	-0.093	-0.161	0.225	∗0.417

**Table 6:** Correlation coefficients of the low-value groups of CT with clinical and serological measures in early RA.

Variable	Disease duration	RF	Anti-CCP	Anti-RA33
Low groups of CT	0.065	0.291	0.120	-0.174
High groups of CT	0.374	0.018	0.029	0.072

**Table 7:** Evaluation of the diagnostic performance using single tests and test combinations.

Variable	Without PCT and CT		With PCT and CT		*p* value
	Sn/Sp	LR+/LR-	Sn/Sp	LR+/LR-	
Early RA vs. all controls					
RF	64.58/61.24	1.67/0.58	89.58/58.99	2.18/0.18	<0.01
Anti-CCP	81.58/76.97	3.54/0.24	92.11/62.92	2.48/0.13	>0.05
Anti-RA33	58.33/74.72	2.31/0.56	91.67/59.55	2.27/0.14	<0.05
RF and anti-CCP	86.84/50.56	1.76/0.26	92.11/62.36	2.45/0.13	<0.05
RF and anti-RA33	47.92/84.27	3.05/0.62	87.5/64.04	2.43/0.20	<0.01
Anti-CCP and anti-RA33	76.32/82.02	4.25/0.29	97.37/58.99	2.37/0.05	>0.05
Early RA vs. disease controls					
RF	64.58/60.20	1.62/0.59	81.25/58.16	1.94/0.32	<0.01
Anti-CCP	81.58/96.94	26.65/0.19	89.47/54.08	1.95/0.19	<0.01
Anti-RA33	58.33/74.79	2.29/0.56	87.50/54.08	1.91/0.23	>0.05
RF and anti-CCP	92.11/66.33	2.74/0.12	89.47/54.08	1.95/0.19	>0.05
RF and anti-RA33	66.67/62.24	1.77/0.54	83.33/58.16	1.99/0.29	<0.01
Anti-CCP and anti-RA33	81.58/91.84	9.99/0.20	86.84/58.16	2.08/0.23	<0.05
Early RA vs. healthy					
RF	33.33/100.00	—^∗^/0.67	83.33/100.00	—^∗^/0.17	<0.01
Anti-CCP	44.74/92.50	5.96/0.60	92.11/93.75	14.74/0.08	<0.01
Anti-RA33	58.33/75.00	2.33/0.56	87.50/91.25	10.00/0.14	<0.01
RF and anti-CCP	57.89/86.25	4.21/0.49	92.11/93.75	14.74/0.08	<0.01
RF and anti-RA33	50.00/96.25	13.33/0.52	83.33/100.00	—^∗^/0.17	<0.01
Anti-CCP and anti-RA33	63.16/78.75	2.97/0.47	92.11/95.00	18.42/0.08	<0.01

^∗^Could not be calculated because the specificity was 100.00. AUC: area under the receiver operating characteristic curve; LR: likelihood ratios; PPV/NPV: positive/negative predictive value; PCT: procalcitonin; CT: calcitonin; RF: rheumatoid factor; anti-CCP: anti-cyclic citrullinated peptide; anti-RA33: anti-RA33 antibodies.

## Data Availability

No data were used to support this study.
